# The hypoxia-related microRNA miR-199a-3p displays tumor suppressor functions in ovarian carcinoma

**DOI:** 10.18632/oncotarget.3604

**Published:** 2015-03-15

**Authors:** Yasuto Kinose, Kenjiro Sawada, Koji Nakamura, Ikuko Sawada, Aska Toda, Erika Nakatsuka, Kae Hashimoto, Seiji Mabuchi, Kazuhiro Takahashi, Hirohisa Kurachi, Ernst Lengyel, Tadashi Kimura

**Affiliations:** ^1^ Department of Obstetrics and Gynecology, Osaka University Graduate School of Medicine, Suita, Osaka, Japan; ^2^ Department of Obstetrics and Gynecology, Yamagata University Faculty of Medicine, Yamagata, Japan; ^3^ Osaka Medical Center and Research Institute for Maternal and Child Health, Izumi, Osaka, Japan; ^4^ Departments of Obstetrics and Gynecology/Section of Gynecologic Oncology, University of Chicago, Chicago, IL, USA

**Keywords:** ovarian cancer, miR-199a-3p, hypoxia, c-Met, microRNA

## Abstract

During the dissemination of ovarian cancer cells, the cells float in the peritoneal cavity without access to a vascular supply and so are exposed to hypoxic conditions, which may cause the ovarian cancer cells to acquire a more aggressive and malignant phenotype. In this study, we screened microRNAs (miRNAs) to identify those that displayed altered expression patterns under hypoxic conditions and then analyzed their functional roles in ovarian cancer progression. miRNA PCR arrays performed on cells from 2 ovarian cancer cell lines (CaOV3 and RMUG-S) revealed miR-199a-3p as one of the miRNAs that are downregulated under hypoxia. *In silico* analyses indicated that *MET* is one of the target genes for miR-199a-3p; subsequently, miR-199a-3p expression was found to be inversely correlated with c-Met expression in ovarian cancer. Transfection of precursor miR-199a-3p into ovarian cancer cells reduced c-Met expression and inhibited the phosphorylation of c-Met, extracellular signal-regulated kinase, and AKT; in addition, proliferation, adhesion, and invasiveness were inhibited. Moreover, overexpression of miR-199a-3p in cancer cells significantly suppressed peritoneal dissemination in a xenograft model. In summary, the hypoxia-related microRNA miR-199a-3p drastically inhibits ovarian cancer progression through the downregulation of c-Met expression. Therefore, miR-199a-3p is a potential target for treating ovarian cancer dissemination.

## INTRODUCTION

Ovarian cancer is the fifth-leading cause of cancer death in women and the most lethal gynecologic malignancy in the Western world [1]. At diagnosis, over 60% of all ovarian cancer patients have advanced disease with peritoneal dissemination and massive ascites, and despite treatments combining aggressive cytoreductive surgery with platinum and taxane-based chemotherapy, the 5-year survival rate at the advanced stages remains only 30% [1]. During ovarian cancer dissemination, cancer cells float in the peritoneal cavity without access to a vascular supply and are thus exposed to hypoxic conditions before they subsequently attach to the peritoneal surface and invade other organs; thus, hypoxia may cause the cells to acquire a more aggressive malignant phenotype [2]. Even though numerous studies on hypoxia and human cancer have been published, the physiological and pathophysiological regulation of hypoxia is still poorly understood [3]. In particular, we lack a clear understanding of how microRNAs (miRNAs) are affected by conditions in the tumor microenvironment, such as hypoxia, although some studies have shown that miRNAs are associated with several key signaling pathways activated by hypoxia and play important roles in hypoxic adaptation [3].

Approximately 22 nucleotides in length, miRNAs are noncoding RNAs that are evolutionarily conserved among different species and are generally involved in posttranscriptional gene regulation. In June 2014, the latest version of the miRNA database miRBase contained 1881 human precursor miRNAs and 2588 mature human miRNAs (miRBase, release 21, June 2014, http://www.mirbase.org/). More than half of the sequences encoding miRNAs are located in cancer-associated genomic regions or fragile sites; thus, miRNAs may regulate cancer at a fundamental level. Therefore, an understanding of miRNAs may contribute to the development of novel approaches to cancer management [4].

In this study, we screened miRNAs to identify those that displayed altered expression patterns under hypoxic conditions and then analyzed their functional roles in ovarian cancer progression. We found that miR-199a-3p is downregulated under hypoxia and that the restoration of this miRNA significantly inhibited ovarian cancer progression. We then investigated mechanisms underlying the possible role of miR-199a-3p in ovarian cancer carcinogenesis and progression.

## RESULTS

### Cells from ovarian cancer cell lines display increased adhesion and invasiveness under hypoxia

**Figure 1:**
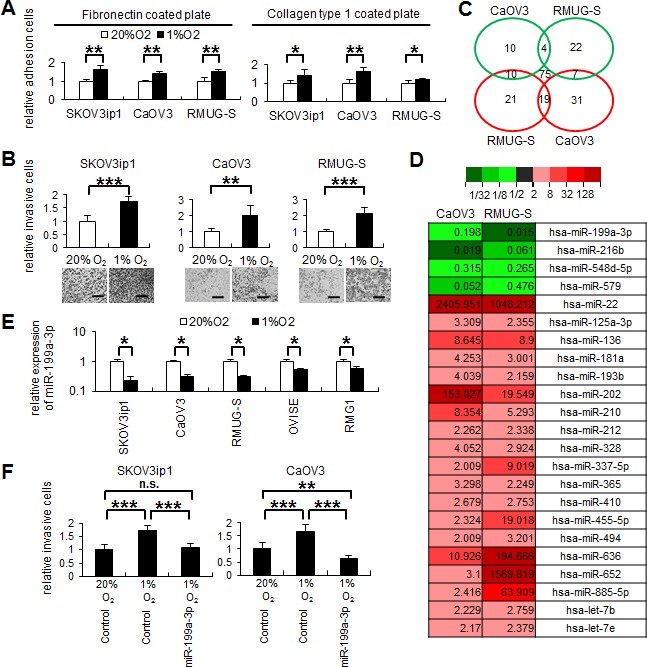
Hypoxia downregulates miR-199a-3p expression, and the restoration of miR-199a-3p inhibits ovarian cancer invasion induced by hypoxia (A) *In vitro* adhesion assay. Ovarian cancer cells (SKOV3ip1, CaOV3, and RMUG-S; 5 × 10^4^ cells) were plated onto 50 μg/mL fibronectin- or collagen type 1–coated 96-well plates under 20% O_2_ or 1% O_2_. After 75 minutes incubation, plates were washed, fixed, and stained with Giemsa solution. The number of adherent cells was counted under a light microscope. Data represent mean ± SEM; n = 5. (B) Matrigel invasion assay. Ovarian cancer cells (5 × 10^4^ cells) were plated in serum-free medium into a modified Boyden chamber system coated with 25 μg Matrigel and the allowed to invade the lower chamber, which contained Dulbecco's modified Eagle's medium supplemented with 10% fetal bovine serum, for 24 hours under 20% O_2_ or 1% O_2_. Noninvading cells were removed with a cotton swab, and invading cells on the underside of the filter were counted. Representative images are shown. Bar represents 200 μm. Data represent mean ± SEM; n = 10. (C) Venn diagrams showing microRNAs (miRNAs) commonly differentially expressed between CaOV3 and RMUG-S cells. CaOV3 and RMUG-S cells were incubated under 20% O_2_ or 1% O_2_ for 48 hours. Total RNA was collected using TRI Reagent and subjected to TaqMan miRNA RT-PCR arrays. Green circles represent miRNAs which expression levels were decreased by <0.5-fold under hypoxia. Red circles show miRNAs which expression levels were increased by >2.0-fold under hypoxia. (D) Heat Map representing color-coded expression levels of 23 miRNAs commonly differentially expressed under hypoxia. (E) Results of the miRNA real-time RT-quantitative PCR. The expression of miR-199a-3p was downregulated under hypoxia in 5 ovarian cancer cells. The 2^−ΔΔCT^ method was used to determine the relative abundance of miR-199a-3p with respect to *RNU6B* expression. (F) Matrigel invasion assay. SKOV3ip1 and CaOV3 cells were transfected with either pre-miR-199a-3p or the negative control miRNA, and allowed to invade for 24 hours. The results show that miR-199a-3p inhibits cell invasion induced by hypoxia. ^*^P < 0.05; ^**^P < 0.01; ^***^P < 0.001; n.s., not significant.

First, we examined the effect of hypoxia on the adhesive and invasive activities of ovarian cancer cells. An *in vitro* cell adhesion assay was performed using 3 ovarian cancer cell lines (SKOV3ip1: serous adenocarcinoma, CaOV3: serous adenocarcinoma, and RMUG-S: mucinous adenocarcinoma) cultured under 20% O_2_ or 1% O_2_. Under hypoxia (1% O_2_), the cells from these lines displayed significantly increased adhesion onto extracellular matrix materials such as fibronectin or collagen type 1 than under normoxia (fibronectin: SKOV3ip1, 1.65-fold increase; CaOV3, 1.41-fold increase; RMUG-S, 1.56-fold increase; collagen type 1: SKOV3ip1, 1.44-fold increase; CaOV3, 1.64-fold increase; RMUG-S, 1.20-fold increase; Figure 1A). Similarly, under hypoxia, the cells displayed significantly increased invasion into Matrigel (SKOV3ip1, 1.73-fold increase; CaOV3, 1.99-fold increase; RMUG-S, 2.13-fold increase; Figure 1B).

### The miRNA miR-199a-3p is downregulated in ovarian cancer cell lines under hypoxia

Since hypoxia significantly promoted the adhesion and invasiveness of ovarian cancer cell lines, we next analyzed the involvement of miRNAs in this process. For this purpose, TaqMan microRNA arrays were performed using CaOV3 or RMUG-S cells to identify miRNAs with altered expression under hypoxia (1% O_2_) (Figure 1C and Supplemental Table 1). In both cell lines, miR-199a-3p, miR-216b, miR-548d-5p, and miR-579 were downregulated in response to hypoxia (i.e., the level of expression under hypoxia was <0.5-fold that under normoxia; Figure 1D), and 19 miRNAs were upregulated in response to hypoxia (i.e., the level of expression under hypoxia was >2.0-fold that under normoxia; Figure 1D and Supplemental Table 1). In this study, we focused our attention on miR-199a-3p, since miR-199a-3p expression was significantly downregulated under hypoxia in all 5 ovarian cancer cell lines examined (SKOV3ip1, CaOV3, RMUG-S, OVISE, and RMG1; Figure 1E).

### Overexpression of miR-199a-3p significantly attenuates the invasiveness of ovarian cancer cell lines under hypoxia

To determine whether miR-199a-3p downregulation was involved in ovarian cancer invasion, scrambled (control) miRNA or the miR-199a-3p precursor was transfected into ovarian cancer cells. High transfection efficacy (approximately 80%) without impairment of cell viability was confirmed by examining cells transfected with FAM-labeled control miRNA under a confocal laser scanning microscope (Supplemental Figure 1). Overexpression of miR-199a-3p significantly attenuated the invasiveness of SKOV3ip1 and CaOV3 cells under hypoxia (Figure 1F); this observation strongly suggests that the downregulation of miR-199a-3p regulates ovarian cancer progression under hypoxia. Since HIF-1α is well-known as the major transcription factor of hypoxia and has been reported to regulate the expression of several miRNAs [3], we transfected an HIF-1α expression vector to ovarian cancer cells under normoxia and assessed miR-199a-3p expression; however, miR-199a-3p expression was not altered by HIF-1α overexpression (Supplemental Figure 2A and 2B), suggesting that alternative pathways other than HIF signaling might be involved in the regulation of miR-199a-3p.

### The miRNA miR-199a-3p is expressed at low levels in ovarian cancer cells

**Figure 2:**
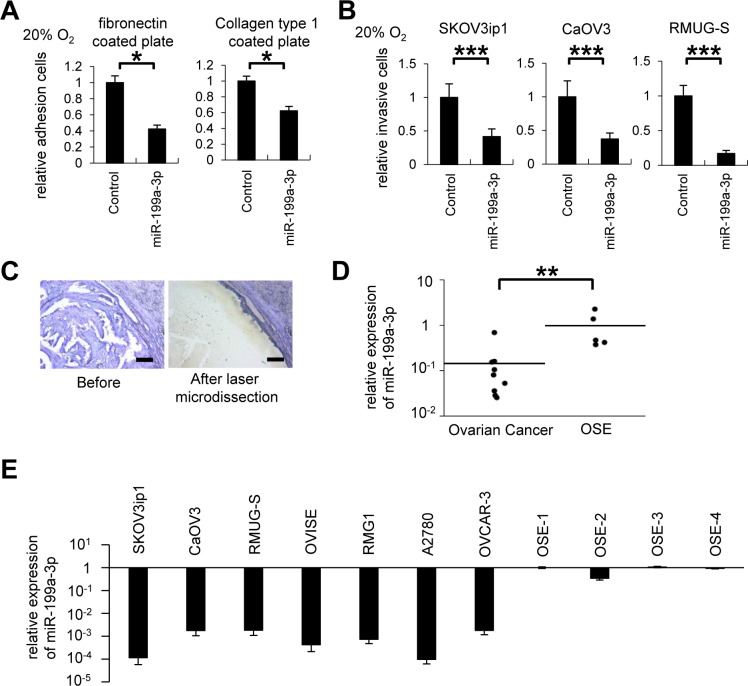
MiR-199a-3p displays tumor suppressor functions, and its expression is downregulated in ovarian cancer specimens and cell lines (A) *In vitro* adhesion assay. SKOV3ip1 cells (5 × 10^4^ cells) were plated onto fibronectin- or collagen type 1–coated 96-well plates under 20% O_2_ for 75 minutes. Data represent mean ± SEM, n = 5. (B) Matrigel invasion assay. Ovarian cancer cells (5 × 10^4^ cells) were plated in a Matrigel-coated upper chamber in serum-free medium and allowed to invade the lower chamber, which contained in 10% fetal bovine serum + Dulbecco's modified Eagle's medium for 24 hours under 20% O_2_ condition. Data represent mean ± SEM, n=7. (C) Representative images of selective collection from formalin fixed paraffin-embedded tissues of ovarian cancer tumor region (high-grade serous adenocarcinoma) using laser capture microdissection. Bar represents 200 μm. (D) miRNA RT-qPCR. Ovarian cancer tumor tissue (high-grade serous adenocarcinoma) or normal ovarian surface epithelium was selectively collected*. The expression of miR-199a-3p was significantly downregulated in ovarian cancer clinical samples.* (E) miRNA RT-qPCR. The expression of miR-199a-3p in 7 ovarian cancer cell lines (SKOV3ip1, CaOV3, RMUG-S, OVISE, RMG1, A2780, and OVCAR-3) was significantly lower than that of OSE (ovarian surface epithelium); ^*^P < 0.05; ^**^P < 0.01; ^***^P < 0.001.

We next investigated whether miR-199a-3p affects ovarian cancer progression under normoxia. Overexpression of miR-199a-3p significantly inhibited adhesion (Figure 2A) and invasiveness (Figure 2B) in ovarian cancer cell lines under normoxia. Since the data indicated that miR-199a-3p downregulation is involved in ovarian cancer invasiveness, we then analyzed miR-199a-3p expression in clinical ovarian cancer samples. For this purpose, formalin fixed paraffin-embedded (FFPE) tissues from 9 patients with high-grade serous carcinoma were collected. The characteristics of these patients are summarized in Supplemental Table 2. Normal ovaries from 5 patients with benign gynecological diseases were used as a control. RNA was extracted from laser-captured ovarian cancer cells, as shown in Figure 2C, and levels of miR-199a-3p expression was examined by miRNA real-time RT-quantitative PCR (RT-qPCR). The expression of miR-199a-3p in the ovarian cancer cells was significantly lower (0.15-fold) than that in normal ovarian surface epithelium (OSE; Figure 2D). Similarly, miR-199a-3p expression in 7 ovarian cancer cell lines (SKOV3ip1, CaOV3, RMUG-S, OVISE, RMG1, A2780, and OVCAR-3) was significantly lower (with relative expression levels ranging from 1.73 × 10^−5^ to 2.76 × 10^−4^) than that in 4 separate OSE cells (Figure 2E). These data suggest that the downregulation of miR-199a-3p might be involved in ovarian carcinogenesis.

### *MET* is a direct target of miR-199a-3p

**Figure 3:**
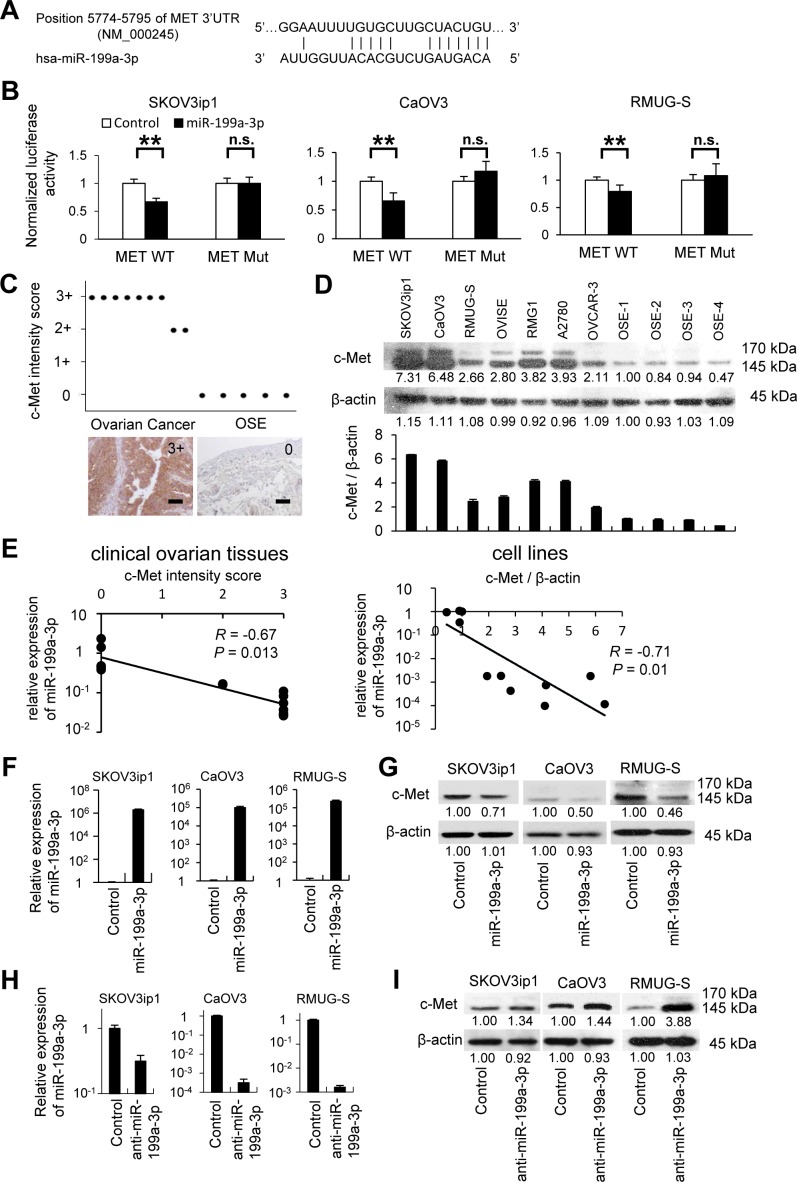
*MET* is a direct target of miR-199a-3p and both are reciprocally expressed in ovarian cancer specimens and cell lines (A) Alignment of potential miR-199a-3p binding site in the 3′-UTR of *MET*. (B) Luciferase assay. Ovarian cancer cells were transfected with a firefly luciferase reporter vector containing the 3′-UTR of *MET* or a mutated 3′-UTR. At 24 hours after the transfection, cells were further transfected with pre-miR-199a-3p or negative control miR. Luciferase activity normalized to the activity of Renilla luciferase was measured. Data represent mean ± SEM, n = 5. (C) Immunohistochemistry. Ovarian cancer tissues and normal ovaries from benign diseases were stained with an antibody against c-Met. c-Met is overexpressed in ovarian cancer clinical tissues. Bar represents 100 μm. (D) Western blotting. c-Met were overexpressed in ovarian cancer cell lines than 4 different OSE (ovarian surface epithelium) cells (upper). Densitometric ratio of the expression of c-Met / β-actin (lower). (E) Correlation plots of miR-199a-3p and c-Met by Pearson's product-moment coefficient. Correlation plot from miR-199a-3p and c-Met expression in ovarian cancer specimens (left) and cell line (right) showing that endogenous miR-199a-3p level is inversely correlated with MET protein levels (*R* = −0.67, *P* = 0.013, *R* = −0.71, *P* = 0.01, respectively). (F) miRNA RT-qPCR. Cells were transfected with pre-miR-199a-3p or negative control miR. Twenty-four hours after transfection, total RNA was collected and subjected to RT-PCR. 2^−ΔΔCT^ method was used to calculate the relative abundance of miR-199a-3p with respect to *RNU6B* expression. Relative fold differences with respect to the control are presented; columns represent the means from 3 independent experiments. (G) Western blotting. Enforced expression of miR-199a-3p inhibits c-Met protein expression in ovarian cancer cell lines. (H) miRNA RT-qPCR. Cells were transfected with anti-miR-199a-3p or negative control miR for 24 hours. (I) Western blotting. Inhibition of miR-199a-3p expression increases c-Met protein expression. **; *P* < 0.01. n.s.; not significant. Densitometry ratios in each western blotting are shown below each blot.

Next, we attempted to determine how miR-199a-3p downregulation is involved in ovarian cancer carcinogenesis. In a previous study, *in silico* analysis revealed a putative miR-199a-3p target site in the 3′-untranslated region (UTR) of *MET* mRNA (bp 5774–5795, NM_000245; Figure 3A) [5]. Thus, we constructed a pMIR-REPORT firefly luciferase miRNA expression reporter vector containing this putative miR-199a-3p binding site for use in luciferase reporter assays. A significant decrease in relative luciferase activity was observed in 3 ovarian cancer cell lines (SKOV3ip1, 0.67-fold; CaOV3, 0.66-fold; and RMUG-S, 0.79-fold; *P* < 0.01) transfected with pre-miR-199a-3p as compared to those transfected with control miRNA (Figure 3B). In addition, site-specific mutation of the target sequence prevented the downregulation of luciferase activity by pre-miR-199a-3p; this result indicates that miR-199a-3p directly interacts with the *MET* 3′UTR.

To determine whether the expression levels of miR-199a-3p and c-Met are inversely correlated in ovarian cancer cells, c-Met expression was assessed by immunohistochemistry using the same FFPE samples as used for the initial characterization of miR-199a-3p expression. Ovarian cancer cells displayed high levels of c-Met expression, whereas OSE was negative for c-Met staining (Figure 3C). Similarly, western blot analysis revealed that ovarian cancer cell lines expressed a high level of c-Met as compared with primary cultured OSE cells from 4 different patients (Figure 3D). A Pearson's product-moment coefficient test showed a significant inverse relationship between miR-199a-3p and c-Met expression in clinical ovarian tissues as well as in cell lines (clinical ovarian tissues, *R*=−0.67, *P*=0.013; cell lines, *R*=−0.71, *P*=0.01, Figure 3E).

In order to determine whether miR-199a-3p regulates c-Met expression, we performed an *in vitro* functional analysis by either restoring or silencing miR-199a-3p. The SKOV3ip1, CaOV3, and RMUG-S cell lines were chosen for the overexpression experiments because they display low constitutive levels of miR-199a-3p miRNA and high c-Met expression. The overexpression of miR-199a-3p in these ovarian cancer cell lines was confirmed by miRNA RT-PCR (Figure 3F). Overexpression of miR-199a-3p caused a significant decrease in c-Met levels, as shown by western blot (Figure 3G). In contrast, inhibition of miR-199a-3p by the transfection of its antagomir induced an increase in c-Met expression (Figure 3H and 3I).

### The miRNA miR-199a-3p inhibits cell proliferation, adhesion, and invasiveness through the suppression of c-Met expression

**Figure 4:**
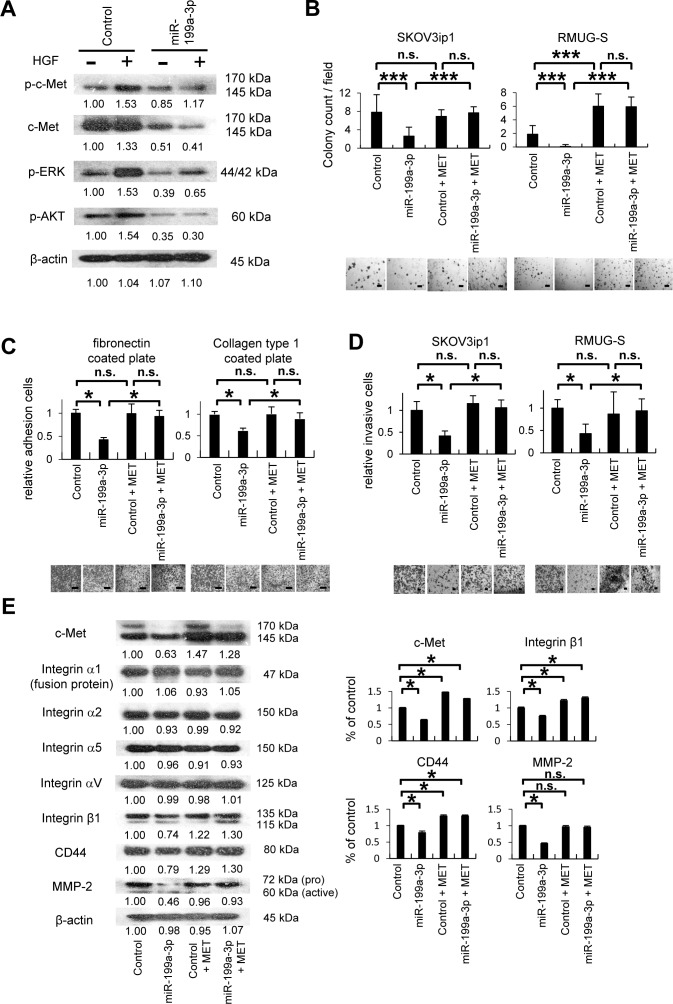
MiR-199a-3p inhibits cell proliferation, adhesion and invasion through the suppression of c-Met expression (A) Western blotting. SKOV3ip1 cells were transfected with pre-miR-199a-3p or negative control miR for 24 hours. Cell lysate were collected before and after HGF stimulation (40 ng/mL, 10 minutes). Immunoblotting was performed with antibodies against phosphorylated c-Met (p-c-Met), c-Met, phosphorylated extracellular signal-regulated kinase (p-ERK), phosphorylated Akt (p-Akt), or β–actin. (B) Colony formation assay. Ovarian cancer cells (1 × 10^5^ cells; SKOV3ip1 and RMUG-S), cotransfected with miRNA (pre-miR-199a-3p or negative control miRNA) and an pIRES2-EGFP vector (containing *MET* or the empty control vector), were suspended in 0.33% agarose. After 10 days, colonies with a diameter greater than 100 μm were counted under a microscope at 40× magnification. Representative images are shown. Bar represents 400 μm. Data represent mean ± SEM; n = 10. (C) *In vitro* adhesion assay. SKOV3ip1 (5 × 10^4^) cells were cotransfected with miRNA (pre-miR-199a-3p or negative control miR) and the pIRES2-EGFP vector (containing *MET* or the empty control vector). The cells were plated onto 50 μg/mL fibronectin- or collagen type 1–coated 96-well plates. After being incubated for 75 minutes at 37°C, the plates were washed to discard nonadherent cells, and the number of adherent cells was counted. Representative images are shown. Bar represents 100 μm. Data represent mean ± SEM; n = 6. (D) Matrigel invasion assay. Ovarian cancer cells were cotransfected with miRNA (pre-miR-199a-3p or negative control miR) and an pIRES2-EGFP vector (containing *MET* or the empty control vector). The cells (5 × 10^4^) were plated in serum-free medium in modified Boyden chamber system that had been coated with 25 μg Matrigel and allowed to invade the lower chamber, which contained Dulbecco's modified Eagle's medium supplemented with 10% fetal bovine serum, for 24 hours. Noninvading cells were removed with a cotton swab, and invading cells on the underside of the filter were counted. Representative images are shown. Bar represents 200 μm. Data represent mean ± SEM; n = 10. (E) Western blotting. SKOV3ip1 cells were cotransfected with miRNA (pre-miR-199a-3p or negative control miR) and an pIRES2-EGFP vector (containing *MET*, or the empty control vector) for 4 hours. Cell lysates were then obtained, and immunoblotting was performed with antibodies against c-Met, various integrins (α1, α2, α5, αV, and β1), CD44, matrix metalloproteinase-2 (MMP-2), and β-actin (left). Densitometric ratios of the expression of c-Met, integrin β1, CD44, and MMP-2 (right). ^*^P < 0.05; ^***^P < 0.001; n.s., not significant. Densitometry ratios in each western blotting are shown below each blot.

HGF/SF stimulation leads to the activation of c-Met via phosphorylation, which leads to the subsequent activation of the MAPK, PI3K, and STAT signaling pathways, resulting in multiple biological effects [6]. We therefore asked whether the signaling pathways activated by HGF/SF stimulation can be inhibited by miR-199a-3p (Figure 4A). In SKOV3ip1 cells transfected with control miRNA, treatment with HGF/SF activated the tyrosine phosphorylation of c-Met. In contrast, transfection with miR-199a-3p almost completely inhibited the phosphorylation of c-Met in response to HGF/SF stimulation. Similarly, in SKOV3ip1 cells transfected with control miRNA, extracellular signal-regulated kinase (ERK) was phosphorylated in response to HGF/SF stimulation, whereas transfection with miR-199a-3p drastically inhibited the phosphorylation of ERK following HGF/SF treatment. To evaluate the activity of the PI3K signaling pathway, AKT phosphorylation in the presence or absence of miR-199a-3p was evaluated. AKT phosphorylation was also inhibited by miR-199a-3p overexpression.

Since miR-199a-3p affects c-Met signaling, we overexpressed miR-199a-3p in ovarian cancer cell lines and then evaluated proliferation, adhesion, and invasiveness in the transfected cells *in vitro*. Further, to restore c-Met expression after the transduction of miR-199a-3p, a full length wild type MET expression vector was co-transfected. Successful transfection was confirmed by western blotting using HEK293T cells as a transfection control, SKOV3ip1, RMUG-S, and OVCAR-3 (Supplemental Figure 3). Overexpression of miR-199a-3p in SKOV3ip1 and RMUG-S cells significantly inhibited anchorage-independent proliferation in soft-agar gel: the numbers of colonies formed by SKOV3ip1 and RMUG-S cells transfected with miR-199a-3p were 0.33-fold and 0.09-fold less, respectively, than those formed by cells transfected with control miRNA (Figure 4B). However, coexpression of c-Met with miR-199a-3p restored cell proliferation levels. In *in vitro* cell proliferation assay, the transfection of miR-199a-3p into SKOV3ip1 and RMUG-S cells significantly impaired cell proliferation (0.66-fold and 0.29-fold less, respectively) and coexpression of c-Met with miR-199a-3p restored cell proliferation levels (Supplemental Figure 4). Overexpression of miR-199a-3p significantly impaired the adhesion of SKOV3ip1 cells onto both fibronectin and collagen type 1: the relative numbers of miR-199a-3p–transfected cells that adhered to fibronectin or collagen were 0.42-fold and 0.62-fold less, respectively, than those of cells transfected with control miRNA (Figure 4C). Whereas, coexpression of c-Met with miR-199a-3p rescued the impaired adhesion ability seen with miR-199a-3p overexpression. In an *in vitro* invasion assay, miR-199a-3p suppressed the invasive ability of SKOV3ip1 and RMUG-S cells: the relative numbers of miR-199a-3p–transfected cells with invasive activity were 0.41-fold and 0.43-fold less, respectively, than those of cells transfected with control miRNA. However, coexpression of c-Met with miR-199a-3p restored the decreased invasive function seen with miR-199a-3p overexpression (Figure 4D).

Next, we used western blotting to examine the molecular changes caused by overexpression of miR-199a-3p or overexpression of miR-199a-3p with c-Met. Overexpression of miR-199a-3p in SKOV3ip1 cells inhibited the expression of integrin β1, CD44, and matrix metalloproteinase-2 (MMP-2), whereas cotransfection with the c-Met expression vector restored the expression of these proteins (Figure 4E). In contrast, the expression of integrins α1, α2, α5, and αV was not altered in response to miR-199a-3p overexpression. These results show that miR-199a-3p regulates proliferation, adhesion, and invasiveness in ovarian cancer cells through the inhibition of c-Met expression and thus affects the expression of downstream proteins such as integrin β1, CD44, and MMP-2.

### Overexpression of miR-199a-3p in SKOV-3-13 cells significantly suppressed peritoneal dissemination in a xenograft model

**Figure 5:**
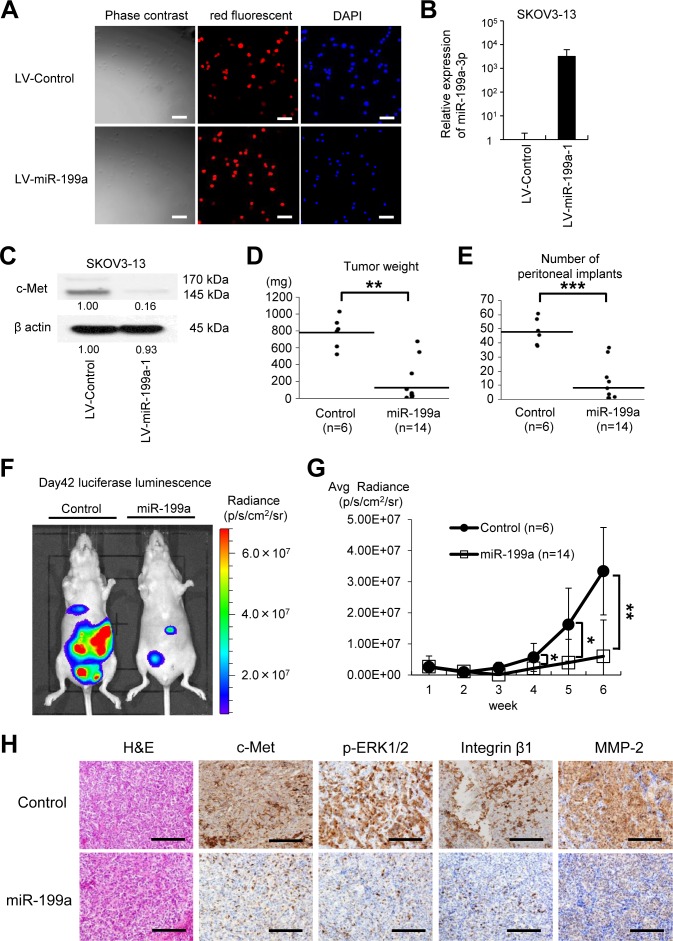
The microRNA miR-199a inhibits peritoneal metastasis in an ovarian cancer xenograft model (A) SKOV-3-13 cells, which stably express the firefly luciferase gene, were transduced with a miR-199a-1 lentivirus or a control microRNA (miRNA) lentivirus and then selected by continuous exposure to 10 μg/mL puromycin. High and stable lentiviral transduction efficiency (more than 90%) was confirmed by the red fluorescent puromycin-N-acetyl-transferase from the Lenti-miRNA-vector (BioSettia); cells are counterstained with DAPI (blue). Bar represents 100 μm. (B) miRNA RT-qPCR. RNA was collected from the lentivirus-transduced SKOV3-13 cells. (C) Western blotting. Overexpression of miR-199a-1 decreases c-Met protein expression. Densitometry ratios in each western blotting are shown below each blot. (D) and (E) Xenograft model. The cells (1 × 10^6^ cells) were injected intraperitoneally (i.p.) into female BALB/c nu/nu mice. Six weeks after the inoculation, mice were sacrificed. Overexpression of miR-199a significantly suppressed tumor growth (D) and the number of peritoneal implants (E). Results are expressed as mean ± SEM; control, n = 6; miR-199a-3p, n = 14. (F) Representative pictures of animals (left, control miRNA; right, miR-199a-1) at 6 weeks after inoculation with SKOV3-13 cells. Pictures were taken using an IVIS imaging system with i.p. administration of 150 mg/kg of D-luciferin potassium. (G) Luciferase activities of peritoneal tumors were measured weekly. Mice that were injected with SKOV3-13 cells overexpressing miR-199a-1 expressed significantly lower luciferase luminescence activities. (H) Immunohistochemical analysis of the peritoneal implants. Representative tumor areas were stained with H&E, antibodies against c-Met, phosphorylated extracellular signal-regulated kinase (p-ERK), integrin β1, and matrix metalloproteinase-2 (MMP-2). Bar represents 200 μm; ^*^P < 0.05; ^**^P < 0.01; ^***^P < 0.001.

As overexpression of miR-199a-3p suppressed proliferation, adhesion, and invasiveness in ovarian cancer cells, we examined the therapeutic potential of miR-199a-3p overexpression in an ovarian cancer xenograft model. Lentiviruses containing miR-199a-3p or a control miRNA were obtained and stably transduced into SKOV-3-13 cells, which stably express the firefly luciferase gene. High and stable transduction efficiency (more than 90%) was confirmed by observation of the red fluorescent puromycin-N-acetyl-transferase expressed by the lentiviral vector (Figure 5A). Overexpression of miR-199a-3p was confirmed by miRNA RT-PCR (Figure 5B) and significantly suppressed c-Met expression, as determined by western blotting (Figure 5C). Six weeks after the intraperitoneal inoculation (1 × 10^6^ cells) of the transduced SKOV-3-13 cells into female BALB/c nu/nu mice, mice showed multiple tumors on the peritoneal surface, omentum, small bowel mesentery, and both ovaries. However, both the total tumor burden (Figure 5D) and the number of metastases (Figure 5E) in mice inoculated with cells overexpressing miR-199a-3p were significantly lesser than those in mice inoculated with cells overexpressing the control miRNA (tumor weight: control, 784.5 ± 169.9 mg vs. miR-199a-3p, 133.2 ± 212.2 mg, *P* < 0.01; number of peritoneal implants: control, 48.3 ± 8.5 vs. miR-199a-3p, 8.4 ± 12.1, *P* < 0.001).

To assess peritoneal tumor progression over time, luciferase activity was measured weekly (Figure 5F and 5G). Four weeks after the inoculation, significant differences were observed in luciferase activity levels (reflecting tumor volume in the peritoneal cavity) between mice that had been injected with cells overexpressing miR-199a-3p and those injected with the control cells. In order to analyze the effect of miR-199a-3p *in vivo*, inoculated tumors were harvested and analyzed by immunohistochemistry (Figure 5H). Overexpression of miR-199a remarkably inhibited c-Met expression *in vivo* as compared with the control, as well as the expressions of downstream molecules such as p-ERK, integrin β1, and MMP-2. These *in vivo* data indicate that ovarian cancer progression is inhibited by miR-199a-3p via the suppression of c-Met and suggest the therapeutic potential of miR-199a-3p in ovarian cancer treatment.

## DISCUSSION

Increasing evidence indicates that miRNAs play important roles in human cancer and thus are promising therapeutic targets [7]. The fact that various miRNAs are remarkably deregulated in ovarian cancer strongly suggests that they are involved in the initiation and progression of this disease [8]. In this study, we found that the miRNA miR-199a-3p was downregulated in ovarian cancer tissues compared with normal ovarian epithelium and that its expression was inversely correlated with the expression of c-Met, a receptor tyrosine kinase that contributes to malignant phenotypes in ovarian cancer. Furthermore, overexpression of miR-199a-3p significantly inhibited proliferation, adhesion, and invasiveness in ovarian cancer cells and downregulated the expression of c-Met *in vitro* and *in vivo*. Thus, the results of this study indicate that miR-199a-3p may be a potential target in ovarian cancer treatment because it causes the inhibition of c-Met, which plays a critical role in ovarian cancer dissemination.

There are two loci that encode the precursors for miR-199a-5p (previously called miR-199a) and miR-199a-3p (previously called miR-199a*) in the human genome: one is on chromosome 1 (miR-199a-2, miRBase Accession MI0000281) and is embedded in the antisense strand of intron 14 of *DNM3*, which encodes dynamin 3; the other is on chromosome 19 (miR-199a-1, miRBase Accession MI0000242) and is embedded in the antisense strand of intron 15 of *DNM2*, which encodes dynamin 2 [9]. This miRNA has different expression patterns and plays opposite roles in different human tumors. For instance, miR-199a-3p is upregulated in patients with gastric cancer or metastatic tissues, and high expression is associated with progression-free survival [10]. In contrast, in colorectal cancer, high miR-199a-3p expression could lead to significantly shorter overall survival of the patients [11]. Similarly, miR-199a-3p is upregulated in esophageal adenocarcinoma [12] and Sézary syndrome (T-cell lymphoma) [13]. However, miR-199a-3p is frequently downregulated in hepatocellular carcinoma, where it targets PAK4, CD44, and mTOR [14, 15, and 16] and displays antiproliferative/tumor suppressor functions. Moreover, the downregulation of miR-199a-3p has been reported in osteosarcoma [17], bladder cancer [18], prostate cancer [19], and papillary thyroid carcinoma [20]. Thus, miR-199a-3p is upregulated as an oncogene in some cases and downregulated as a potential tumor suppressor in others. Concerning ovarian cancer, Iorio et al. reported that miR-199a-5p is downregulated in ovarian cancer tissues/cell lines as compared to normal tissues [21]. Nam et al. reported miR-199a significantly correlated with a poor prognosis of serous ovarian cancer [22]. Chen et al. reported that miR-199a-5p is involved in tumor progression and chemoresistance in ovarian cancer by regulating IKKβ expression [23]. In addition, miR-199a-5p also targets *CD44* to suppress tumorigenicity and multidrug resistance in ovarian cancer initiating cells [24]. Recently, Joshi et al reported that *DNM 2* and miRNA-199a-5p reciprocally regulate hypoxia-inducible factor-1α (HIF-1α) and HIF-2 and inhibit ovarian cancer metastasis [25]. Interestingly, miR-199a-3p and miR-199a-5p behave differently and have separate targets, probably due to their different seed regions. Gu et al. mentioned in their review that miR-199a-3p is deregulated primarily during tumorigenesis and hepatitis, whereas miR-199a-5p appears be related to cardiomyocyte function and hypoxia [9]. In this study, by using laser-captured ovarian cancer tissues and ovarian cancer cell lines, we showed that the expression of miR-199a-3p is drastically decreased compared with that in normal ovarian epithelium; this observation suggests that miR-199a-3p is substantially involved in ovarian cancer tumorigenesis.

We also showed that hypoxia downregulates miR-199a-3p expression. Hypoxia is known to induce changes in the expression of a number of miRNAs (termed hypoxamirs) and is considered to be an important proximal regulator of miRNA biogenesis and function [26]. While HIF-1α, a major transcription factor of hypoxia, has been reported to regulate several hypoxamirs [3], the overexpression of HIF-1α did not alter the expression of miR-199a-3p in ovarian cancer cells as shown in Supplemental Figure 2. Other genes or signaling pathways contribute to the adaptation of tumor cells to hypoxia. For instance, hypoxia could promote the induction of miR-21 *via* an Akt2-dependent process in mouse mammary adenocarcinomas and human ovarian carcinomas [27]. Wu C, et al. reported that hypoxia increases type I collagen prolyl-4-hydroxylase [C-P4H(I)], which leads to prolyl-hydroxylation and accumulation of Argonaute2 (Ago2), a critical component of the RNA-induced silencing complex (RISC) [28]. Such a mechanism might be involved in the regulation of miR-199a-3p under hypoxia. Further elucidation will be needed to clarify how the cellular program of hypoxia regulates miR-199a-3p expression.

Here, we found that c-Met and miR-199a-3p are reciprocally expressed in high-grade serous ovarian carcinomas. Further, we showed that overexpression of miR-199a-3p inhibited c-Met expression and consequently inhibited MAP kinase and PI3-kinase signaling. Overexpression of miR-199a-3p also inhibited anchorage-dependent cell proliferation, adhesion to different extracellular matrix components, and invasion of ovarian cancer cells, paralleled by a significant reduction in the expression of integrin β1, CD44, and MMP2, all of which play pivotal roles in ovarian cancer progression. Since the overexpression of c-Met expression almost completely abrogated the inhibitory effects induced by miR-199a-3p, c-Met inhibition appears to be crucial for the anti-proliferative/tumor suppressor functions of miR-199a-3p in ovarian cancer, although a miRNA usually has hundreds of target genes. Usually, c-Met expression is very low in normal tissues, but it is dysregulated in many types of carcinomas as a result of gene amplification, activating mutations, dimerization from overexpression, and autocrine or paracrine stimulatory mechanisms [29]. With regard to ovarian cancer, others and we have reported that the overexpression of c-Met is an independent prognostic factor for overall survival, and its inhibition could reduce cancer cells adhesion, invasiveness, and metastasis [30]. Other reports have shown that miR-199a-3p targets *MET* in several cancer cell lines [5, 16, and 31]. In clinical cancer tissues, Minna et al. recently showed that, similar to our findings, miR-199a-3p and c-Met were reciprocally expressed in papillary thyroid carcinoma [20].

Since the HGF-MET axis is frequently dysregulated in many types of cancer and MET dysregulation is associated with an increased propensity for metastatic disease and poor overall prognosis [32], HGF/MET inhibition has emerged as a target for anticancer therapies. Thus far, a number of monoclonal antibodies to HGF and MET, as well as small molecule inhibitors of MET, are currently being developed. Preclinically, several cancer progressions, including non-small cell lung cancer, hepatocellular carcinoma, and gastric cancer, have been attenuated by MET inhibition. Furthermore, ongoing clinical trials with tivantinib, cabozantinib, onartuzumab, crizotinib, rilotumumab, and ficlatuzumab have shown encouraging results [33]. However, none have reached to Phase III clinical trials regarding ovarian cancer, suggesting that the mere inhibition of MET might not be sufficient to overcome this miserable disease. miR-199a-3p is significantly down-regulated in ovarian cancer and its restoration has been reported to be linked with the inhibition of PAK4, CD44, and mTOR [14, 15, and 16], all of which are related to cancer progression, indicating that miR-199a-3p might have multi-tumor suppressor functions at least in ovarian cancer. Besides, because miRNAs are endogenously produced and have higher stability and better tissue compatibility than other agents, they might be more effective than currently available therapeutics [19]. With these reasons, we assume miR-199a-3p can be a potential ideal therapeutic target for ovarian cancer. The keys for miRNA drug development are that the chemical structure must be stable *in vivo*, cell permeable, and should hybridize to the target organ of interest with high specificity and affinity [34]. Recently, locked nucleic acid–antimir-122 (miravirsen) successfully entered phase II trials for the treatment of hepatitis C virus (HCV) infection [35]. Miravirsen caused a dose-dependent reduction in HCV RNA levels, and dose-limiting adverse effects or escape mutations in the miR-122 binding sites of the HCV genome were not observed. This approach may facilitate a rapid route for miRNA replacement therapy in the clinical setting in the near future.

**Figure 6:**
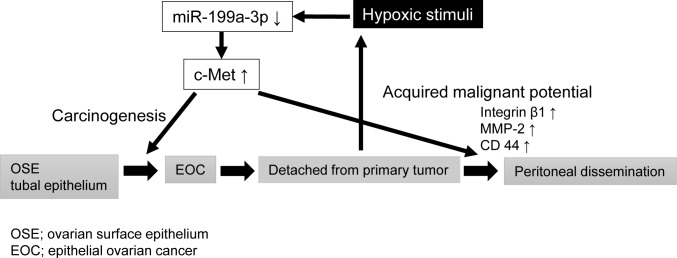
Schematic diagram showing the potential role of miR-199a-3p in ovarian cancer carcinogenesis and progression

In conclusion, in the present study, we found that miR-199a-3p is downregulated under hypoxia and that miR-199a-3p and c-Met are reciprocally expressed in ovarian cancer tissues. We further identified this hypoxia-related miRNA as a suppressor of ovarian cancer peritoneal dissemination via the inhibition of c-Met expression. Thus, we conclude that the loss of miR-199a-3p is deeply involved in ovarian carcinogenesis by leading to the upregulation of c-Met. In addition, when ovarian cancer cells disseminate into the peritoneal cavity, hypoxia further downregulates miR-199a-3p expression and thus allows cancer cells to acquire more aggressive phenotypes (Figure 6). Although there is still a long way to go before the advent of an effective and nontoxic miRNA-based cancer therapy, targeting miR-199a-3p might open a new approach for ovarian cancer treatment and should be explored further for future clinical application.

## MATERIALS AND METHODS

### Materials

Dulbecco's modified Eagle's medium (DMEM; #08458-45) was obtained from Nacalai Tesque (Kyoto, Japan). Fetal bovine serum (FBS; #172012) and hepatocyte growth factor (HGF; #H1404) were purchased from Sigma Aldrich (St. Louis, MO, USA). Human fibronectin (#356008), collagen type 1 (#354236), growth factor reduced basement membrane proteins (Matrigel, #356230), and the Integrins Sampler Kit [#611435; includes monoclonal antibodies against integrin α2; #611016, α5; #610633, αV; #611012, and β1; #610467] were purchased from BD Biosciences (Franklin Lakes, NJ, USA). Antibodies against c-Met (C-28; #161), integrin α1 (R-164; #10728), and MMP-2 (4D3 and H-76; #53630 and #10736, respectively) were obtained from Santa Cruz Biotechnology (Dallas, TX, USA). Antibodies against β-actin (#4967), phospho-AKT (Ser473; #9271), phospho-ERK 1/2 (p-ERK1/2; Thr202/Tyr204, E10; #9106), and CD44 (156-3C11; #3570) were obtained from Cell Signaling (Danvers, MA, USA). Anti-phospho-c-Met antibody (pYpYpY^1230/1234/1235^; #44888G), Lipofectamine 2000 Transfection Reagent (#11668027), Lipofectamine 3000 Reagent (#L3000008) and TRIzol Reagent (#15596-018) were from Life Technologies (Carlsbad, CA, USA). The anti–c-Met antibody (EP1454Y; #51067) was from Abcam (Cambridge, UK).

### Cell culture

SKOV3ip1 cell line was generously provided by Dr. Gordon B. Mills (M.D. Anderson Cancer Center, Houston, TX, USA). CaOV3 and A2780 cell lines were purchased from ATCC (Manassas, VA, USA). RMUG-S, OVISE, and RMG1 cell lines were obtained from the Health Science Research Resources Bank (Osaka, Japan). OVCAR-3 cell line was provided by the Cell Resource Center for Biomedical Research Institute of Development, Aging and Cancer of Tohoku University (Sendai, Japan). SKOV-3 cells with stable expression of the beta subunit of human gonadotropin (β-hCG) and firefly luciferase were previously generated and named as SKOV-3-13 [36]. HEK293T cells were used as a transfection control and were kindly gifted from Dr. Keiichi Kumasama (Osaka University, Suita, Osaka, Japan). Cells were cultured in DMEM supplemented with 10% FBS and 1000 U/mL penicillin/streptomycin and incubated in 95% air/5% CO_2_ at 37°C.

### Adhesion assay

Ovarian cancer cells (5 × 10^4^ cells) were plated onto 50 μg/mL fibronectin- or collagen type 1–coated 96-well plates under 20% O_2_ or 1% O_2_. After 75 minutes of incubation, the plates were washed, fixed, and stained with Giemsa solution. The number of adherent cells was counted under a light microscope.

### Matrigel invasion assay

Ovarian cancer cells (5 × 10^4^ cells) were plated in in serum-free medium in modified Boyden chamber system that had been coated with 25 μg of Matrigel and allowed to invade the lower chamber, which contained DMEM supplemented with 10% FBS, for 24 hours. Noninvading cells were removed with a cotton swab, and the number of invading cells on the underside of the chamber was counted.

### The miRNA RT-qPCR array

CaOV3 or RMUG-S cells were incubated under 20% O_2_ or 1% O_2_ for 48 hours. Then, total RNA was extracted using TRIzol Reagent and subjected to miRNA RT-qPCR. The miRNA expression profiling was performed using the stem loop RT-qPCR–based TaqMan® Human MicroRNA Array Set v2.0 (Applied Biosystems, Carlsbad, CA USA; #4398965) according to the manufacturer's protocol.

### RT-qPCR analysis of miR-199a-3p

RT-qPCR was performed using the StepOnePlus Real-Time PCR System (Applied Biosystems, Foster City, CA, USA). Total RNA was extracted using TRIzol and was transcribed into cDNA by using the TaqMan MicroRNA Reverse Transcription Kit (Applied Biosystems; #4366596). Mature miR-199a-3p was assayed using a TaqMan assay (hsa-miR-199a-3p; #002304). The TaqMan endogenous control (*RNU6B*; #001093) was used to normalize miRNA expression levels. Comparative real-time PCR was performed in triplicate, and relative levels of miR-199a-3p expression were calculated using the 2^−ΔΔCt^ method [37].

### Transfection of miRNA

Ovarian cancer cells were transfected with precursor miRNA (pre-hsa-miR-199a-3p, #PM11779) or inhibitor miRNA (anti-hsa-miR-199a-3p, #AM11779) at a concentration of 166 nM. Pre-miR miRNA Precursor Negative Control #1 (#AM17110) was used as a control, and FAM-labeled Pre-miR Negative Control #1 (#AM17121) was used to confirm the transfection efficacy. All oligonucleotides were purchased from Life Techonologies. Oligonucleotide transfection was performed using Lipofectamine 2000 in accordance with the manufacturer's instructions. Twenty-four hours after transfection, cells were collected for subsequent analysis. Transfection efficacy was confirmed by detecting FAM-labeled cells using a FV1000-D Laser Scanning Confocal Microscope (Olympus, Tokyo, Japan).

### MicroRNA sampling from FFPE tissue samples

Total RNA was isolated from FFPE tissue slides (10 μm thick) by using the miRNeasy FFPE Kit (Qiagen, Venlo, The Netherlands; #217504) according to the manufacturer's protocol. Ovarian cancer tumor tissue (high-grade serous adenocarcinoma) or normal OSE was selectively collected using laser capture microdissection (LMD7000, Leica Microsystems, Wetzlar, Germany).

### Luciferase assay

Synthetic oligonucleotides with 4 copies of the *MET* 3′-UTR (GAATTTTGTGCTTGCTACTGTAT; bp 5774–5795 of NM_000245), which were predicted to bind hsa-miR-199a-3p, or 4 copies of a mutated version of the sequence (GAATTTAGTCCTTGCTAGTGAAT) that contained *Mlu*Ι and *Xho*Ι restriction sites were cloned into the pMIR-REPORT firefly luciferase miRNA expression reporter vector (Applied Biosystems; #AM5795). After 5 × 10^4^ ovarian cancer cells were seeded in 24-well plates, 0.5 μg of the pMIR-REPORT vector and 0.05 μg of the pRL-TK Renilla luciferase control vector (Promega, Madison, WI, USA; #E2241) were cotransfected using Lipofectamine 2000. Twenty-four hours after the transfection, the cells were further transfected with 150 pM pre-miR-199a-3p or the negative control miRNA; 24 hours later, luciferase activity was measured using the Dual-Luciferase Reporter Assay System (Promega; #E1910) according to the manufacturer's instructions. Firefly luciferase activities were normalized to Renilla luciferase activities.

### Immunohistochemistry

The slides were deparaffinized in xylene and rehydrated with 100% ethanol before antigen unmasking was performed by boiling the slides in Target Retrieval Solution (pH 9.0; Dako, Glostrup, Denmark; #S2367). After being blocked with Dako REAL Peroxidase-Blocking Solution (Dako; #S2023), the slides were incubated with primary antibodies against c-Met (Abcam; #51067) at 1:400, p-ERK1/2 (Cell Signaling; #9106) at 1:200, integrin β1 at 1:200 (BD Biosciences; #610467), and MMP-2 at 1:200 (Santa Cruz; #53630) for 1 hour at room temperature. After washing with PBS containing 0.01% Tween-20, the slides were stained using the EnVision System (Dako; #K4002) and then counterstained with Carrazzi's hematoxylin. The c-Met staining was scored based on the staining intensity (0, none; 1+, weak; 2+, medium; and 3+, strong).

### Western blotting

A total of 1 × 10^5^ cells was plated into a 6-well plate and then lysed with 1× Cell Lysis Buffer (Cell Signaling; #9803). Lysates (15 μg) were separated by SDS-PAGE using SuperSep Ace, 5–20% (Wako Pure Chemical Industries, Osaka, Japan; #197-15011) and transferred to polyvinylidene difluoride membranes (GE Healthcare, Little Chalfont, UK; #10600029). The membranes were then incubated with the primary antibodies (1:1000 in 5% BSA) and then with a corresponding secondary horseradish peroxidase conjugated IgG. The proteins were visualized with an electrochemiluminescent system (PerkinElmer, Waltham, MA; #NEL105001EA).

### Co-transfection of miRNA and MET expression vector

pIRES2-EGFP empty vector (5.3kb) and pIRES2-EGFP wt-Met vector (11.9kb) were generously gifted from Dr. Patrick Ma (University of Chicago, Chicago, IL). Full length wild type-MET (6641 bp; NM_000245) was inserted into *EcoR*I site. Ovarian cancer cells were co-transfected with pre-hsa-miR-199a-3p (#PM11779) or pre-miR miRNA Precursor Negative Control #1 (#AM17100) at a concentration of 166 nM, and pIRES2-EGFP vector or pIRES2-EGFP wt-Met vector at a concentration of 2.5 μg/mL using Lipofectamine 3000 in accordance with the manufacturer's instructions. Four hours after the transfection, cells were washed with serum-free DMEM three times to reduce toxicity and were cultured in DMEM supplemented with 10% FBS. 24 hours later, cells were used for subsequent analysis. Transfection efficacy (more than 80%) was confirmed by detecting GFP-positive cells using fluorescence microscope.

### *In vitro* colony formation assay

Ovarian cancer cells (1 × 10^5^ cells) were suspended in 0.33% agarose and seeded into a 6-well plate containing a basal layer of 0.5% agarose. After 10 days, colonies greater than 100 μm in diameter were counted under a microscope at 40× magnification.

### Lentivirus transduction

The lentivirus vectors, hsa-mir-199a-1 lentivirus (#mir-LV156) or mir-control lentivirus (#mir-LV000), were obtained from BioSettia (San Diego, CA, USA) at a titer of 10^7^ IU/mL. For the *in vivo* experiments, SKOV-3-13 cells, which stably express firefly luciferase [36], were used as follows: 1 × 10^5^ cells were plated in 6-well plates overnight, and then 20 μL of lentivirus diluted in 2 mL of fresh DMEM medium supplemented with 10% FBS was added in the presence of 5 μg/mL of polybrene (EMD Millipore, Billerica, MA, USA; #TR-1003-G). Twenty-four hours later, the culture medium was replaced with fresh medium, and the transduced cells were positively selected by continuous exposure to 5 μg/mL puromycin (InvivoGen, San Diego, CA, USA; #ant-pr-1). Fourteen days after the selection, more than 90% of the cells displayed red fluorescence at excitation/emission wavelengths of 587/610 nm.

### Xenograft model

Female athymic BALB/c nude mice (aged 5 to 6 weeks) were purchased from CLEA Japan Inc. (Tokyo, Japan) and were maintained under aseptic conditions at constant humidity and temperature (25–28°C). All animal experiments were approved by the Institutional Animal Care and Use Committee of Osaka University, in accordance with institutional and NIH guidelines. The SKOV-3-13 cells (1 × 10^6^ cells) stably expressing either miR-199a-1 or control miRNA were injected intraperitoneally (i.p.) into female athymic nude mice. Mice were assessed daily for general health condition. For bioluminescence imaging of living animals, mice were injected i.p. with 150 mg/kg D-luciferin potassium salt (Wako; #126-05114) in PBS, anesthetized with 2.0% isofluorane, and imaged (exposure time 1–300 seconds; binning, medium; field of view, 10 × 10 cm; F/stop 1; open filter; anterior side) by IVIS Lumina II (Caliper, Hopkinton, MA, USA) once a week. For analysis, regions of interest were drawn for each peritoneal cavity, and the average radiance (p/s/cm^2^/sr) was measured using Living Imaging 3.0 software (Caliper LifeSciences). Six weeks after inoculation, the mice were sacrificed, the number of metastases in each mouse was counted, and the tumors carefully dissected and weighed. Tumor tissues were immediately fixed and embedded in paraffin. The slides were prepared and immunohistochemical analysis with antibodies against c-Met, p-ERK1/2, integrin β1, or MMP-2 were performed as previously described.

### *In vitro* cell proliferation assay

The cells (3 × 10^3^ cells of SKOV3ip1 or 6 × 10^3^ cells of RMUG-S) were seeded onto 96-well plates and cultured in DMEM supplemented with 10% FBS for 4 days. The relative number of viable cells was assessed using the CyQUANT cell proliferation assay kit (Life Technologies, #C7026) according to the manufacturer's instructions.

### Statistical analysis

Statcel version 3 (OMS-Publishing Inc., Saitama, Japan) and JMP version 10.0.2 (SAS Institute Japan Ltd., Tokyo, Japan) were used for statistical analysis. Data except for RT-qPCR are expressed as mean ± SEM. In RT-qPCR data, the error bars represent standard deviation calculated by the StepOnePlus Real-Time PCR System. Differences were analyzed using the Mann-Whitney U test. To analyze correlation between two variables, the Pearson's product-moment coefficient of correlation and associated *P* value were calculated. Differences were considered statistically significant at *P* < 0.05.

## SUPPLEMENTARY METERIALS, FIGURES AND TABLES


