# Supplementary material for: The hypoxia-related microRNA miR-199a-3p displays tumor suppressor functions in ovarian carcinoma
Source: Oncotarget. 2015 Mar 15;6(13):11342–56. doi: 10.18632/oncotarget.3604 (PMC4484460; doi:10.18632/oncotarget.3604)
Supplement: Supplementary file 1 [file oncotarget-06-11342-s001.pdf]

The hypoxia-related microRNA miR-199a-3p displays tumor suppressor functions in ovarian carcinoma

Supplementary Material

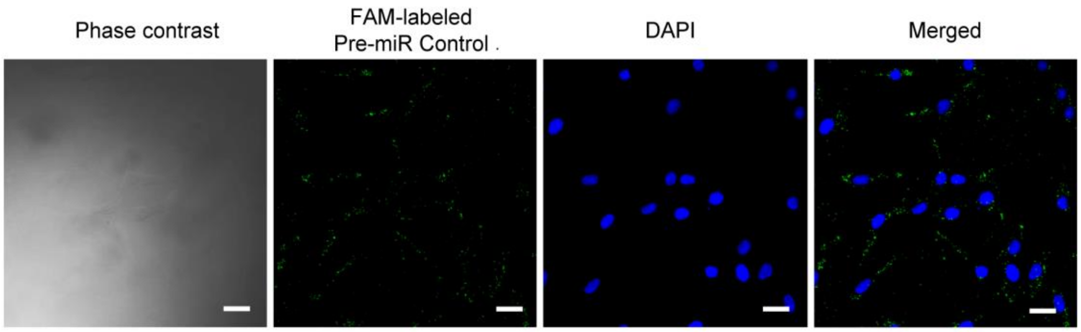

Supplemental Figure 1: High transfection efficacy without impairment of cell viability was confirmed by using a confocal laser scanning microscope to observe cells transfected with FAM-labeled control microRNA (green) and stained with DAPI (blue). Bar represents 50 µm.

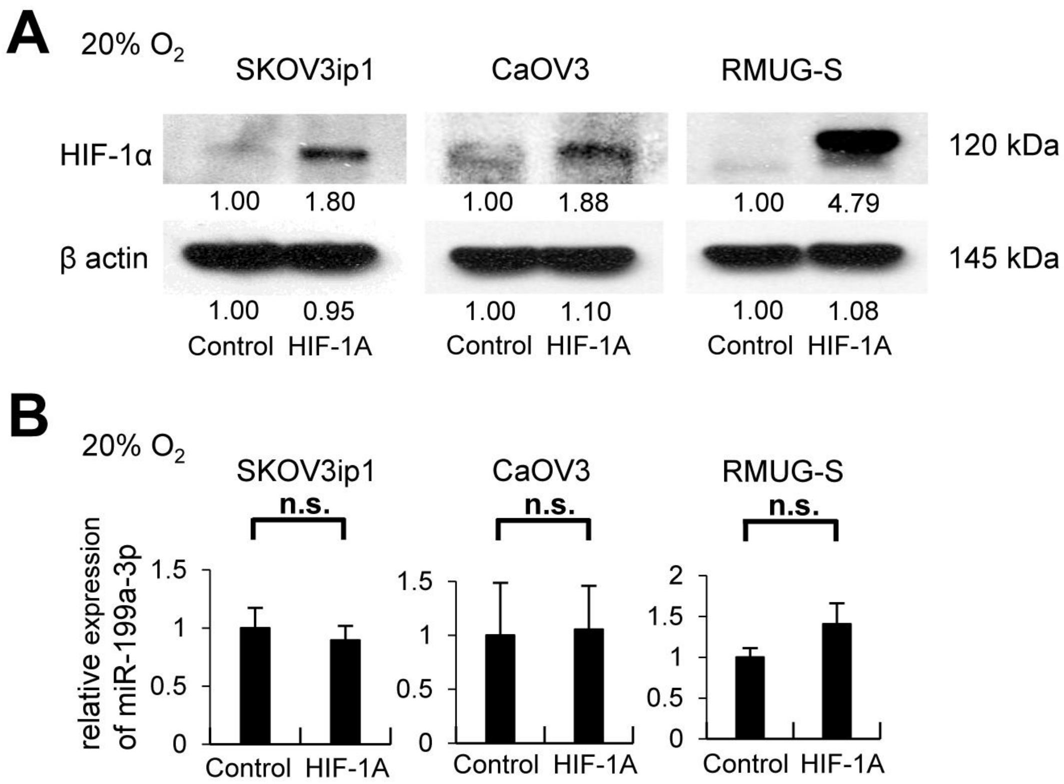

Supplemental Figure 2: (A) Ovarian cancer cells were transfected with a HIF-1α expression vector for 24 hours under 20% O_2. Cell lysates were collected and subjected to western blotting with an antibody against HIF-1α. (B) miRNA RT-qPCR. Overexpression of HIF-1α did not influence miR-199a-3p expression. Densitometry ratios in each western blotting are shown below each blot. n.s., not significant.

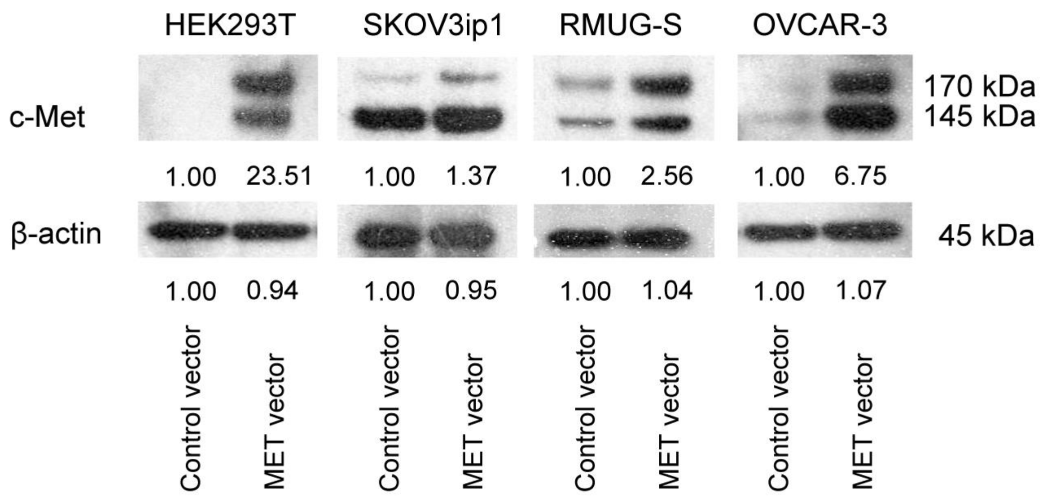

Supplemental Figure 3: Western blotting. Cells (HEK293T, SKOV3ip1, RMUG-S, and OVCAR-3 were transfected with an pIRES2-EGFP vector (containing *MET* or the empty control vector). Cell lysates were collected and immunoblotted with antibodies against c-Met and β-actin. Densitometry ratios in each western blotting are shown below each blot.

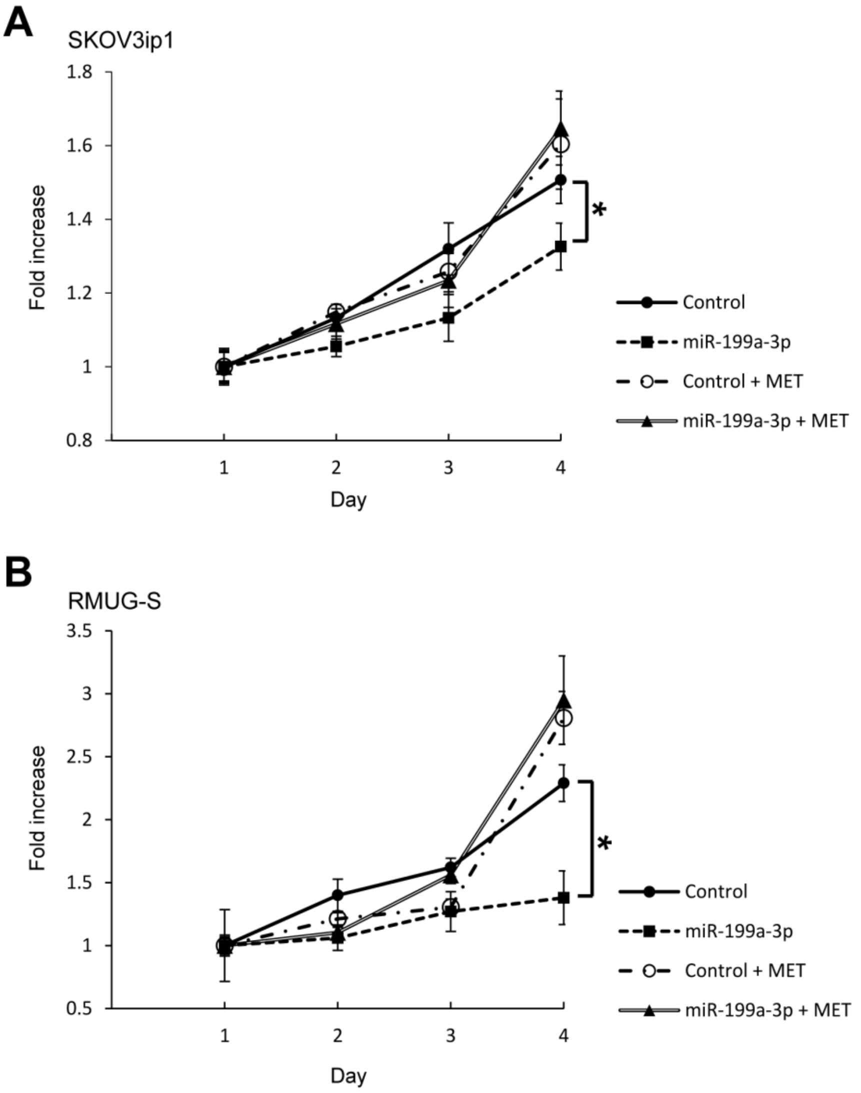

Supplemental Figure 4: *In vitro* cell proliferation assay. SKOV3ip1 (A) or RMUG-S (B) cells were cotransfected with miRNA (pre-miR-199a-3p or negative control miR) and an pIERS2-EGFP vector (containing *MET*, or the empty control vector). The cells (3×10^3 cells of SKOV3ip1 or 6×10^3 cells of RMUG-S) were seeded onto 96-well plates and cultured 1in DMEM supplemented with 10% FBS for 4 days. Relative viable cell number was assessed using the CyQUANT cell proliferation assay kit (Life Technologies) according to the manufacturer's instructions. Data represent mean ± SEM; n = 10 from triplicate independent experiments. $^{*}P < 0.05$.

Supplemental Table 1: the results of miRNA RT-PCR array. Relative expression levels of miRNAs in CaOV3 and RMUG-S cells under hypoxia as compared to normoxia are shown. N/A, not available.

	CaOV3	RMUG-S
hsa-let-7a	1.388	2.033
hsa-let-7b	2.229	2.759
hsa-let-7c	2.073	0.768
hsa-let-7d	1.556	1.637
hsa-let-7e	2.170	2.379
hsa-let-7f	1.952	0.878
hsa-let-7g	1.625	1.669
hsa-miR-1	N/A	N/A
hsa-miR-100	1.065	1.076
hsa-miR-101	0.911	1.945
hsa-miR-103	2.046	0.932
hsa-miR-105	N/A	N/A
hsa-miR-106a	0.977	0.427
hsa-miR-106b	1.517	0.944
hsa-miR-107	1.160	1.949
hsa-miR-10a	0.589	0.337
hsa-miR-10b	1.227	3.542
hsa-miR-122	N/A	N/A
hsa-miR-124	N/A	N/A
hsa-miR-125a-3p	3.309	2.355
hsa-miR-125a-5p	1.542	0.740
hsa-miR-125b	1.954	1.453
hsa-miR-126	2.263	0.856
hsa-miR-127-3p	1.659	N/A
hsa-miR-127-5p	N/A	N/A
hsa-miR-128	0.349	0.929
hsa-miR-129-3p	6.721	N/A
hsa-miR-129-5p	38.217	N/A
hsa-miR-130a	1.246	1.984
hsa-miR-130b	1.491	1.664
hsa-miR-132	2.133	0.802
hsa-miR-133a	12.352	N/A
hsa-miR-133b	17.674	N/A
hsa-miR-134	2.131	N/A
hsa-miR-135a	2.651	0.502
hsa-miR-135b	1.365	0.823
hsa-miR-136	8.645	8.900

hsa-miR-137	1.404	0.397
hsa-miR-138	0.934	0.425
hsa-miR-139-3p	N/A	N/A
hsa-miR-139-5p	1.795	0.479
hsa-miR-140-3p	1.094	0.586
hsa-miR-140-5p	1.190	0.705
hsa-miR-141	1.891	1.128
hsa-miR-142-3p	1.358	N/A
hsa-miR-142-5p	N/A	N/A
hsa-miR-143	N/A	18.633
hsa-miR-145	N/A	0.018
hsa-miR-146a	1.804	3.278
hsa-miR-146b-3p	19.149	N/A
hsa-miR-146b-5p	1.280	1.156
hsa-miR-147	N/A	N/A
hsa-miR-147b	N/A	N/A
hsa-miR-148a	1.543	1.214
hsa-miR-148b	1.089	0.852
hsa-miR-149	1.713	0.331
hsa-miR-150	17.163	0.227
hsa-miR-152	1.356	1.406
hsa-miR-153	N/A	N/A
hsa-miR-154	N/A	N/A
hsa-miR-155	N/A	8.572
hsa-miR-15a	0.033	0.645
hsa-miR-15b	1.111	0.930
hsa-miR-16	1.513	0.632
hsa-miR-17	1.198	0.459
hsa-miR-181a	4.253	3.001
hsa-miR-181c	5.782	1.879
hsa-miR-182	2.954	1.193
hsa-miR-183	2.684	1.248
hsa-miR-184	N/A	3.432
hsa-miR-185	1.713	0.821
hsa-miR-186	1.413	0.493
hsa-miR-187	N/A	N/A
hsa-miR-188-3p	N/A	N/A
hsa-miR-18a	0.950	0.435
hsa-miR-18b	0.616	0.549
hsa-miR-190	0.393	1.223
hsa-miR-191	1.474	0.580
hsa-miR-192	1.528	1.205
hsa-miR-193a-3p	1.853	0.491

hsa-miR-193a-5p	1.231	2.350
hsa-miR-193b	4.039	2.159
hsa-miR-194	1.897	1.883
hsa-miR-195	5.078	0.709
hsa-miR-196b	1.776	4.115
hsa-miR-197	1.960	1.128
hsa-miR-198	N/A	N/A
hsa-miR-199a-3p	0.198	0.015
hsa-miR-199a-5p	N/A	N/A
hsa-miR-199b-5p	N/A	N/A
hsa-miR-19a	1.214	0.571
hsa-miR-19b	0.900	0.625
hsa-miR-200a	1.355	1.301
hsa-miR-200b	1.809	1.107
hsa-miR-200c	1.740	0.823
hsa-miR-202	153.027	19.549
hsa-miR-203	1.515	0.590
hsa-miR-204	1.090	18.879
hsa-miR-205	1.290	0.670
hsa-miR-208	N/A	N/A
hsa-miR-208b	N/A	N/A
hsa-miR-20a	0.980	0.877
hsa-miR-20b	0.908	0.565
hsa-miR-21	1.690	2.353
hsa-miR-210	8.354	5.293
hsa-miR-211	37.055	N/A
hsa-miR-212	2.262	2.338
hsa-miR-214	79.014	N/A
hsa-miR-215	N/A	1.504
hsa-miR-216a	18.941	N/A
hsa-miR-216b	0.019	0.061
hsa-miR-217	N/A	N/A
hsa-miR-218	2.616	1.805
hsa-miR-219-1-3p	0.037	N/A
hsa-miR-219-2-3p	N/A	N/A
hsa-miR-219-5p	N/A	N/A
hsa-miR-22	2405.951	1048.212
hsa-miR-220	N/A	N/A
hsa-miR-220b	N/A	N/A
hsa-miR-220c	N/A	N/A
hsa-miR-221	1.236	0.773
hsa-miR-222	1.176	1.140
hsa-miR-223	1.199	2.040

hsa-miR-224	1.027	0.619
hsa-miR-23a	1.010	1.913
hsa-miR-23b	1.121	2.009
hsa-miR-24	2.218	0.724
hsa-miR-25	1.145	0.634
hsa-miR-26a	1.540	1.751
hsa-miR-26b	1.849	1.332
hsa-miR-27a	1.970	0.781
hsa-miR-27b	1.834	0.731
hsa-miR-28-3p	1.355	0.817
hsa-miR-28-5p	N/A	0.756
hsa-miR-296-3p	N/A	N/A
hsa-miR-296-5p	19.338	0.892
hsa-miR-298	N/A	N/A
hsa-miR-299-3p	N/A	N/A
hsa-miR-299-5p	0.925	N/A
hsa-miR-29a	1.535	1.417
hsa-miR-29b	1.274	1.906
hsa-miR-29c	1.814	2.005
hsa-miR-301a	2.050	1.041
hsa-miR-301b	1.761	3.449
hsa-miR-302a	N/A	9.350
hsa-miR-302b	N/A	N/A
hsa-miR-302c	N/A	N/A
hsa-miR-30b	1.240	1.396
hsa-miR-30c	1.634	0.712
hsa-miR-31	1.962	0.676
hsa-miR-32	3.151	0.979
hsa-miR-320	1.678	0.900
hsa-miR-323-3p	1.504	0.284
hsa-miR-324-3p	N/A	1.033
hsa-miR-324-5p	N/A	1.106
hsa-miR-325	N/A	N/A
hsa-miR-326	N/A	0.018
hsa-miR-328	4.052	2.924
hsa-miR-329	N/A	N/A
hsa-miR-330-3p	4.581	0.434
hsa-miR-330-5p	N/A	0.717
hsa-miR-331-3p	1.576	0.817
hsa-miR-331-5p	1.054	0.487
hsa-miR-335	0.542	N/A
hsa-miR-337-5p	2.009	9.019
hsa-miR-338-3p	1.547	0.037

hsa-miR-339-3p	1.832	0.397
hsa-miR-339-5p	1.208	0.335
hsa-miR-33b	N/A	N/A
hsa-miR-340	1.488	0.956
hsa-miR-342-3p	2.023	0.877
hsa-miR-342-5p	N/A	N/A
hsa-miR-345	1.330	0.411
hsa-miR-346	N/A	N/A
hsa-miR-34a	1.599	0.996
hsa-miR-34c-5p	3.325	0.253
hsa-miR-361-5p	1.835	0.838
hsa-miR-362-3p	5.877	1.024
hsa-miR-362-5p	2.365	0.278
hsa-miR-363	1.078	0.530
hsa-miR-365	3.298	2.249
hsa-miR-367	N/A	N/A
hsa-miR-369-3p	N/A	4.767
hsa-miR-369-5p	9.200	N/A
hsa-miR-370	7.766	N/A
hsa-miR-371-3p	N/A	N/A
hsa-miR-372	0.143	1.251
hsa-miR-373	0.059	9.404
hsa-miR-374a	1.419	1.233
hsa-miR-374b	1.388	1.112
hsa-miR-375	1.579	0.883
hsa-miR-376a	1.160	4.746
hsa-miR-376b	0.071	N/A
hsa-miR-376c	1.667	0.834
hsa-miR-377	N/A	N/A
hsa-miR-379	1.281	N/A
hsa-miR-380	0.077	N/A
hsa-miR-381	N/A	N/A
hsa-miR-382	2.558	N/A
hsa-miR-383	N/A	N/A
hsa-miR-384	N/A	N/A
hsa-miR-409-5p	N/A	N/A
hsa-miR-410	2.679	2.753
hsa-miR-411	2.194	N/A
hsa-miR-412	N/A	N/A
hsa-miR-422a	1.127	0.395
hsa-miR-423-5p	3.081	1.697
hsa-miR-424	0.993	0.286
hsa-miR-425	1.891	0.962

hsa-miR-429	1.590	0.669
hsa-miR-431	1.915	N/A
hsa-miR-433	2.634	N/A
hsa-miR-448	N/A	N/A
hsa-miR-449a	21.862	1.121
hsa-miR-449b	N/A	2.585
hsa-miR-450a	0.071	0.802
hsa-miR-450b-3p	N/A	N/A
hsa-miR-450b-5p	0.062	70.771
hsa-miR-451	0.075	N/A
hsa-miR-452	2.123	1.007
hsa-miR-453	N/A	N/A
hsa-miR-454	1.286	0.567
hsa-miR-455-3p	74.288	N/A
hsa-miR-455-5p	2.324	19.018
hsa-miR-483-5p	11.370	1.560
hsa-miR-484	0.760	0.647
hsa-miR-485-3p	1.629	0.035
hsa-miR-485-5p	N/A	N/A
hsa-miR-486-3p	4.162	N/A
hsa-miR-486-5p	2.403	N/A
hsa-miR-487a	1381.517	0.001
hsa-miR-487b	1.657	N/A
hsa-miR-488	N/A	N/A
hsa-miR-489	1.087	1.119
hsa-miR-490-3p	N/A	N/A
hsa-miR-491-3p	N/A	N/A
hsa-miR-491-5p	1.605	0.018
hsa-miR-492	N/A	N/A
hsa-miR-493	2.344	N/A
hsa-miR-494	2.009	3.201
hsa-miR-495	2.357	N/A
hsa-miR-496	N/A	N/A
hsa-miR-499-3p	N/A	N/A
hsa-miR-499-5p	N/A	38.008
hsa-miR-500	1.187	0.287
hsa-miR-501-3p	N/A	N/A
hsa-miR-501-5p	9.308	0.468
hsa-miR-502-3p	14.059	0.348
hsa-miR-502-5p	18.656	1.135
hsa-miR-503	15.399	0.879
hsa-miR-504	N/A	N/A
hsa-miR-505	N/A	N/A

hsa-miR-506	N/A	N/A
hsa-miR-507	N/A	N/A
hsa-miR-508-3p	N/A	N/A
hsa-miR-508-5p	N/A	N/A
hsa-miR-509-3-5p	N/A	N/A
hsa-miR-509-5p	N/A	N/A
hsa-miR-510	N/A	N/A
hsa-miR-511	0.081	9.585
hsa-miR-512-3p	19.547	1.611
hsa-miR-512-5p	N/A	1.237
hsa-miR-513-5p	N/A	N/A
hsa-miR-515-3p	N/A	2.790
hsa-miR-515-5p	N/A	2.619
hsa-miR-516a-5p	N/A	0.524
hsa-miR-516b	N/A	9.022
hsa-miR-517a	N/A	3.578
hsa-miR-517b	N/A	9516.386
hsa-miR-517c	0.074	3.056
hsa-miR-518a-3p	N/A	1.286
hsa-miR-518a-5p	N/A	19.197
hsa-miR-518b	39.221	1.723
hsa-miR-518c	N/A	4.831
hsa-miR-518d-3p	N/A	1.034
hsa-miR-518d-5p	N/A	1.163
hsa-miR-518e	18.682	1.851
hsa-miR-518f	N/A	1.458
hsa-miR-519a	4.059	1.393
hsa-miR-519c-3p	N/A	3.709
hsa-miR-519d	N/A	1.267
hsa-miR-519e	0.030	1.408
hsa-miR-520a-3p	N/A	1.673
hsa-miR-520a-5p	N/A	2.050
hsa-miR-520b	N/A	1.589
hsa-miR-520d-5p	N/A	74.251
hsa-miR-520e	N/A	0.571
hsa-miR-520f	N/A	2.831
hsa-miR-520g	N/A	1.800
hsa-miR-521	N/A	1.440
hsa-miR-522	3.193	1.246
hsa-miR-523	0.046	2.318
hsa-miR-524-5p	N/A	N/A
hsa-miR-525-3p	N/A	1.161
hsa-miR-525-5p	N/A	1.206

hsa-miR-526b	N/A	2.265
hsa-miR-532-3p	0.497	0.830
hsa-miR-532-5p	1.049	0.827
hsa-miR-539	1.132	N/A
hsa-miR-541	N/A	N/A
hsa-miR-542-3p	4.507	1.298
hsa-miR-542-5p	0.048	9.321
hsa-miR-544	N/A	N/A
hsa-miR-545	2.862	0.532
hsa-miR-548a-3p	N/A	N/A
hsa-miR-548a-5p	N/A	N/A
hsa-miR-548b-3p	N/A	0.000
hsa-miR-548b-5p	0.136	11.319
hsa-miR-548c-3p	N/A	N/A
hsa-miR-548c-5p	0.026	3.489
hsa-miR-548d-3p	N/A	0.200
hsa-miR-548d-5p	0.315	0.265
hsa-miR-551b	N/A	N/A
hsa-miR-556-3p	N/A	N/A
hsa-miR-556-5p	N/A	N/A
hsa-miR-561	0.116	N/A
hsa-miR-570	N/A	N/A
hsa-miR-574-3p	2.007	1.335
hsa-miR-576-3p	1.422	1.040
hsa-miR-576-5p	N/A	0.040
hsa-miR-579	0.052	0.476
hsa-miR-582-3p	0.025	3.128
hsa-miR-582-5p	0.073	1.177
hsa-miR-589	0.054	3.828
hsa-miR-590-5p	1.728	0.426
hsa-miR-597	0.347	4.910
hsa-miR-598	N/A	1.246
hsa-miR-615-3p	N/A	N/A
hsa-miR-615-5p	1.706	N/A
hsa-miR-616	18.401	N/A
hsa-miR-618	0.139	N/A
hsa-miR-624	N/A	N/A
hsa-miR-625	1.474	2.249
hsa-miR-627	0.141	0.801
hsa-miR-628-5p	2.134	1.911
hsa-miR-629	0.528	216.819
hsa-miR-636	10.926	194.666
hsa-miR-642	0.874	58.662

hsa-miR-651	N/A	0.000
hsa-miR-652	3.100	1569.819
hsa-miR-653	N/A	4.194
hsa-miR-654-3p	1.401	N/A
hsa-miR-654-5p	N/A	N/A
hsa-miR-655	1.196	0.000
hsa-miR-660	1.024	3.939
hsa-miR-671-3p	1.821	36.293
hsa-miR-672	0.073	N/A
hsa-miR-674	N/A	N/A
hsa-miR-708	1.125	9.597
hsa-miR-744	1.922	0.353
hsa-miR-758	2.082	N/A
hsa-miR-871	N/A	N/A
hsa-miR-872	N/A	N/A
hsa-miR-873	N/A	N/A
hsa-miR-874	N/A	N/A
hsa-miR-875-3p	N/A	N/A
hsa-miR-876-3p	19.315	N/A
hsa-miR-876-5p	1.137	N/A
hsa-miR-885-3p	N/A	N/A
hsa-miR-885-5p	2.416	63.909
hsa-miR-886-3p	N/A	N/A
hsa-miR-886-5p	N/A	N/A
hsa-miR-887	N/A	N/A
hsa-miR-888	N/A	4.880
hsa-miR-889	0.599	N/A
hsa-miR-890	N/A	N/A
hsa-miR-891a	N/A	N/A
hsa-miR-891b	N/A	N/A
hsa-miR-892a	N/A	N/A
hsa-miR-9	1.440	0.537
hsa-miR-92a	0.998	0.860
hsa-miR-93	1.073	0.613
hsa-miR-95	1.680	2.019
hsa-miR-96	1.881	2.290
hsa-miR-98	4.463	1.262
hsa-miR-99a	1.061	1.292
hsa-miR-99b	1.496	134.692
hsa-miR-885-5p	2.416	63.909
hsa-let-7b	2.229	2.759
hsa-let-7e	2.170	2.379

Supplemental Table 2: Patient characteristics.

	Normal ovary (control)	Ovarian cancer (high-grade serous adenocarcinoma)
Number of patients	5	9
Median age in years (range)	57.8 (44–64)	54.7 (29–77)
Number of premenopausal patients (%)	1 (20)	4 (44)
Number of postmenopausal patients (%)	4 (80)	5 (55)
Disease		
Number of patients with myoma (%)	3 (60)	
Number of patients with CIN3 (%)	1 (20)	
Number of patients with unilateral ovarian fibroma (%)	1 (20)	
Number of patients with high-grade serous adenocarcinoma (%)		9 (100)
FIGO stage		
Number at stage IIc (%)		1 (11)
Number at stage IIIb (%)		1 (11)
Number at stage IIIc (%)		7 (77)
Residual tumor		
Number of tumors ≤1 cm (%)		8 (88)
Number of tumors >1 cm (%)		1 (11)
Chemotherapy		
Number of patients treated with paclitaxel + carboplatin (%)		9 (100)

CIN3; Cervical intraepithelial neoplasia 3
